# Critical Factors for the Recycling of Different End-of-Life Materials: Wood Wastes, Automotive Shredded Residues, and Dismantled Wind Turbine Blades

**DOI:** 10.3390/polym11101604

**Published:** 2019-10-01

**Authors:** Rachele Castaldo, Francesca De Falco, Roberto Avolio, Emilie Bossanne, Felipe Cicaroni Fernandes, Mariacristina Cocca, Emilia Di Pace, Maria Emanuela Errico, Gennaro Gentile, Dominik Jasiński, Daniele Spinelli, Sonia Albein Urios, Markku Vilkki, Maurizio Avella

**Affiliations:** 1 National Research Council of Italy, Institute for Polymers Composites and Biomaterials, Via Campi Flegrei 34, 80078 Pozzuoli (NA), Italy; rachele.castaldo@ipcb.cnr.it (R.C.); francesca.defalco@ipcb.cnr.it (F.D.F.); roberto.avolio@cnr.it (R.A.); mariacristina.cocca@cnr.it (M.C.); emilia.dipace@cnr.it (E.D.P.); mariaemanuela.errico@cnr.it (M.E.E.); maurizio.avella@cnr.it (M.A.); 2 Institut technologique FCBA,10 rue Galilée, 77420 Champs-sur-Marne, France; emilie.bossanne@fcba.fr; 3 Exergy Ltd, Coventry Innovation Village, CUTP, Cheetah Road, Coventry CV1 2TL, UK; fcicaroni@exergy.uk.com (F.C.F.);; 4 Next Technology Tecnotessile, Via del Gelso 13 Prato, Italy; chemtech@tecnotex.it; 5 Aimplas, Istituto Tecnologico del Plastico, València Parc Tecnològic, Calle Gustave Eiffel 4, 46980 Paterna, Spain; salbein@aimplas.es; 6 Conenor Ltd, Kaitilantie 30, 16300 Orimattila, Finland; markku.vilkki@conenor.com

**Keywords:** recycling, municipal bulky waste, end-of-life vehicles, characterization, FTIR, evolved gas analysis

## Abstract

Different classes of wastes, namely wooden wastes, plastic fractions from automotive shredded residues, and glass fiber reinforced composite wastes obtained from dismantled wind turbines blades were analyzed in view of their possible recycling. Wooden wastes included municipal bulky wastes, construction and demolition wastes, and furniture wastes. The applied characterization protocol, based on Fourier transform infrared (FTIR) spectroscopy in attenuated total reflection (ATR) mode, scanning electron microscopy coupled with energy dispersive X-ray spectroscopy (SEM/EDX), and thermogravimetric analysis (TG) coupled with FTIR spectrometry for the investigation of the evolved gases, revealed that the selected classes of wastes are very complex and heterogeneous materials, containing different impurities that can represent serious obstacles toward their reuse/recycling. Critical parameters were analyzed and discussed, and recommendations were reported for a safe and sustainable recycling of these classes of materials.

## 1. Introduction

Recycling is widely recognized as one of the fundamental steps towards a Circular Economy regenerative system, able to minimize resource input and waste, emission, and energy consumption [1]. In the last years, several classes of materials have been the subject of researches to find environmental sustainable and cost-effective processes for their recycling. These classes include wooden waste [2,3,4,5], plastic fractions from end-of-life vehicles (ELV) [6,7,8,9], and fiber reinforced polymers (FRP) from different sources, such as wind turbine blades [10,11], and boats [12,13].

Amongst them, the use of wood [14,15,16] and FRP as fillers in polymer composites [17,18], and the reprocessing of polymer fractions from ELV have been widely investigated [19]. 

Nevertheless, only in recent years safety factors have gained a growing research interest, with significant studies aimed to underline the presence of harmful substances in waste materials and to prevent their emission during the reprocessing/recycling of specific classes of wastes.

For instance, up to 51 critical substances, including mineral oils, phthalates, phenols, and parabens have been identified in relation to waste paper for recycling [20]. Similarly, mineral oil hydrocarbons, phthalates, phenols, polychlorinated biphenyls, and toxic metals (such as cadmium, cobalt, chromium, copper, nickel, and lead) were identified and quantified in paper and board fractions of municipal solid waste [21].

As concerning wood waste, the analysis of contaminants performed so far in view of their reuse/recycling has been mainly devoted to the identification of metal contaminants (arsenic, chromium, zinc, nickel and copper, lead, mercury, and cadmium) [22] and their quantification, that prevent in many cases their reuse in polymer composites. The analysis of emission of gaseous pollutants from wood has been mainly related to indoor air quality and working environments [23,24] and it has been focused on formaldehyde and ammonia release, but only slightly related to the possible risks associated to the evolution of harmful substances during the recycling of end-of-life (EOL) wooden products.

For ELV, after the dismantling procedure, the so called hulks are transported to a shredder where they are crushed and ground. The metallic and non-metallic parts are separated by various physical and chemical means to be recycled. The residual part is called automotive shredder residue (ASR) and it mainly consists of a mixture of fabrics, fibers, glass, rubber, plastics, composites, dirt, and wood [25]. Currently, these residues are destined to landfilling or energy recovery and only a few analytical investigations have been reported so far aimed at evaluating the composition of ASR in view of these EOL possibilities [26,27].

Glass fiber reinforced polymers (GFRP) are a class of EOL materials for which different recycling processes have been proposed. These processes include thermolysis [28] or pyrolysis [29] and recovery of glass fibers for their recycling in new polymer or ceramic composites, pyrolysis and recovery of the liquid phase (suitable for reuse as petrol and fuel oils), and the gas phase (mainly CO and CO_2_) [30,31]. Moreover, other methods are based on the milling of GFRP for the obtainment of millimeter and micrometer sized particles, and their reuse as fillers in new composite materials [32]. Nevertheless, while each step of the process has been widely investigated, including the energetic aspects related to the milling process [33], characterization techniques have been mainly devoted to the analysis of the obtained products [30,34] and to the effect of the milled GFRP properties (particle size, moisture content, resin content) on the final properties of the new materials realized [35]. The environmental and safety aspects analyzed for GFRP are mainly those related to the analysis of different hazards due to abandoned GFRP items (such as EOL boats) [36], including the inhalation of fiberglass [37,38] and to exposure of workers to monomers used during GFRP production [39]. Nevertheless, the analysis of the composition and the thermal behavior of GFRP in view of their recycling is still missing.

Starting from this scenario, in this work we applied an analytical protocol to evaluate the suitability of the above identified classes of wastes, wood wastes, ASR from ELV, and glass fiber reinforced polymers (GFRP), in view of their possible recycling at the conditions used for the processing of polymers. The analytical protocol set up included the identification of the nature of the main constituents of the waste samples and of the contaminants through Fourier-Transform Infrared (FTIR) analysis, followed by morphological and elemental analysis performed by scanning electron microscopy (SEM) coupled with energy dispersive X-ray (EDX) spectroscopy. Finally, thermogravimetric (TG) analysis in air flow combined to FTIR analysis of the evolved gases was carried out on all the samples using a TG-FTIR apparatus to evaluate the possible evolution of harmful substances during high temperature treatments typical of the processing conditions of thermoplastic polymers and composites.

## 2. Materials and Methods 

### 2.1. Materials

The first class of wooden wastes are municipal bulky waste, kindly provided by TOMRA Gmbh (Mülheim-Kärlich, Germany). This class was constituted by large wooden pieces, with irregular shape and weight. These pieces were coded as MBW-Px, where x identified each specific piece. About 2 kg of these wooden pieces were milled by means of a Retsch (Haan, Germany) SM100 cutting mill equipped with a 2 mm diameter hole bottom sieve. The obtained sample was coded as MBW.

The second class of wooden samples was kindly provided by Conenor Ltd (Orimattila, Finland). The sample, already shredded and with particle size <2 mm, was obtained by milling construction and demolition wastes. The sample was coded CDW.

The third class of wooden samples was kindly provided by the eco-organism Valdelia (Labege, France). Wooden waste was obtained from used professional furniture and it was shredded into 1 mm particles before characterization. This furniture waste sample was coded as FW.

ASR samples recovered from the shredding of ELV were collected by Bellver PLA (Xàtiva - Valencia, Spain) on their industrial plants. ELV samples were obtained using the following process. EOL vehicles were brought to scrapyard where they were decontaminated and deregistered, before being dismantled, smashed, and shredded into irregular pellets of about 3 mm size. This lightweight fraction was constituted by different plastics and a miscellaneous of foam, textiles, rubber, glasses, and tiny copper threads. The presence of different materials in this type of waste represents a serious limitation for their recycling. The mixed stream of ELV with 3 mm granulometry, was treated through an air separator equipped with a zig-zag-shaped sifter channel obtaining a “light” and a “heavy” fraction. As a second step, a metal separator (Sesotec Rapid Vario-FS equipment, city, country) was used to remove metal contaminants. The light and heavy fractions obtained were coded as HF-ELV LF-ELV, respectively. 

The last class of materials, for which different EOL scenarios are the subject of recent researches [40,41], was constituted by wind turbine blade (WTB) wastes, mainly constituted by GFR. Milled WTB waste samples were kindly provided by Conenor Ltd (Orimattila, Finland).

### 2.2. Methods

Attenuated Total Reflectance Fourier-Transform InfraRed (ATR-FTIR) spectroscopy analysis was carried out on wooden samples (MBW, CDW, FW), on ELV samples (ELV, HF-ELV, LF-ELV), and on the WTB sample. A Perkin Elmer (Waltham, MA, USA) Spectrum One FTIR spectrometer equipped with a Universal ATR accessory was used, setting 64 scans and a resolution of 4 cm^−1^, over the range 4000–700 cm^−1^. Before the analysis, samples were conditioned at 25 °C and 50% relative humidity (RH) for at least 24 h.

All the samples were also analyzed by SEM coupled to EDX analysis, to investigate the morphology and to analyze the elemental composition of the selected waste materials in view of their recycling. In particular, SEM analysis was performed using a FEI (Eindhoven, The Netherlands) Quanta 200 FEG environmental SEM (ESEM) in low vacuum mode (P_H2O_ = 80 Pa), using a Large Field Detector (LFD) and an accelerating voltage ranging between 15 and 20 kV. Before the analysis, samples were mounted on aluminum stubs by means of carbon adhesive disks. EDX analysis was performed on the above described SEM using an Oxford Instruments (Abingdon-on-Thames, UK) Inca Energy System 250 and an Inca-X-act LN2-free analytical silicon drift detector. EDX analysis was carried out using the point selection mode or the area selection mode. The accelerating voltage was fixed to 30 kV for EDX analysis. For analysis carried out in the area-selection mode, average results and standard deviation values are based on at least five consecutive measurements on different areas of the samples.

TG analysis in air flow combined to FTIR analysis of the evolved gases was carried out on all the samples using a TG-FTIR apparatus. In particular, evolved gas (EG) analysis was performed using a thermogravimetric analyser Perkin Elmer Pyris 1 coupled to a Perkin Elmer (Waltham, MA, USA) Spectrum™ Frontier spectrometer by a TL 8000 transfer line with a 10 cm gas cell. The transfer line and gas cell were heated to 270 °C to avoid condensation of organic compounds. Approximately 5 mg of each sample were placed in a platinum crucible and heated at a rate of 10 °C/min over a range of 50–800 °C. The purge gases through the TGA was air at a flow rate of 25 mL/min with a balance purge of 45 mL/min. This combined rate of 70 mL/min was kept constant through the transfer line and cell. The FTIR was configured to continuously collect background-corrected spectra over a wave number range of 4000–650 cm^−1^ for the complete duration of the temperature program at a resolution of 4 cm^−1^.

Soxhlet extraction procedure were applied on ELV samples using chloroform as a solvent. The Soxhlet extraction was carried out for 12 h. Then, the samples were dried in oven at 60 °C overnight under vacuum and weighted to quantify the amount of extracted materials. The CHCl_3_ soluble fractions were then characterized by means of ATR-FTIR spectroscopy.

## 3. Results and Discussion

### 3.1. Wooden Samples

#### 3.1.1. MBW Samples

**Figure 1:**
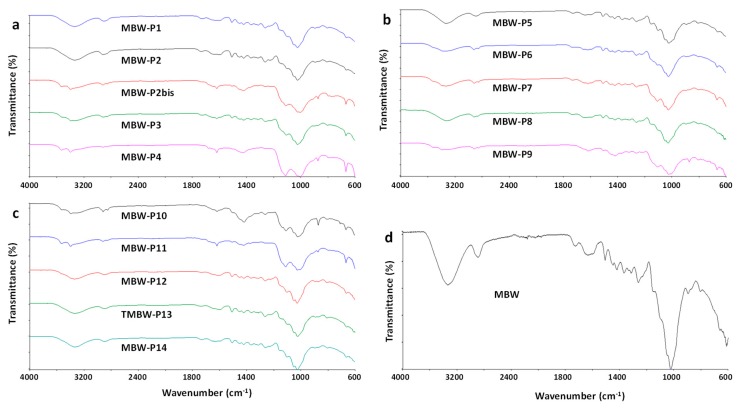
Attenuated total reflectance (ATR)-FTIR spectra of MBW samples.

ATR-FTIR analysis was performed on each individual MBW-Px piece and on the milled, homogenized MBW powder, as well as on milled CDW and FW. FTIR analysis of all the analyzed wooden pieces, MBW-P1-MBW-P14 are reported in Figure 1a–c. The recorded spectra clearly demonstrate the lignocellulosic nature of all the samples, with a strong and complex absorption band in the region 900–1200 cm^−1^, typical of the cellulose fraction [42]. Several samples showed the presence of polymeric binders, possibly urea-formaldehyde (UF) resins, as demonstrated by adsorption bands typical of C=O stretching of amides centred at 1635–1660 cm^−1^, and N–H stretching centred at 3340–3360 cm^−1^ (MBW-P2bis, MBW-P3, MBW-P4, MBW-P7, MBW-P9, MBW-P11) [43,44]. It is to be remarked that the possible presence of inorganic contaminants, especially silicate contaminants, would be masked by the wide absorption bands typical of the lignocellulosic fraction in the wavenumber range 900–1200 cm^−1^ [45,46]. The grinding and homogenization of the samples hided the presence of non-wood fractions, whose occurrence was not clearly detectable in the ATR-FTIR spectrum of milled MBW sample (Figure 1d).

**Figure 2:**
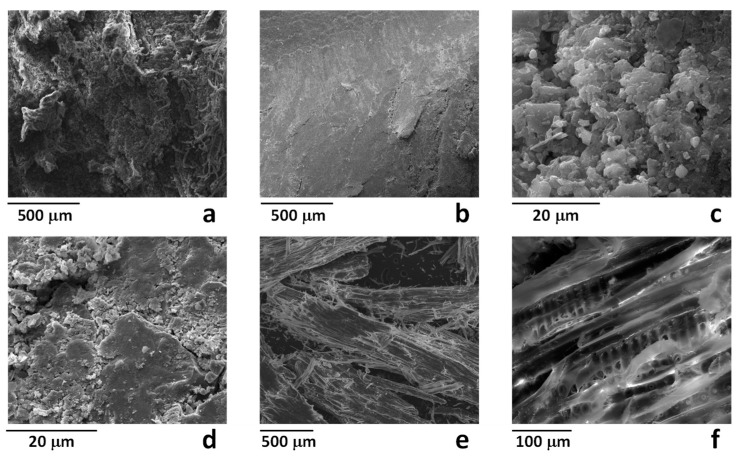
SEM images of the samples MBW-P1 (**a,b**) MBW-P4 (**c,d**) and MBW (**e,f**).

Representative SEM images of MBW-Px samples are reported in Figure 2. Figure 2a shows the typical complex morphology of a wood structure, whereas Figure 2b evidenced the diffuse presence of a compact layer on some areas of the samples. This surface layer, also evidenced at higher magnification in Figure 2c,d seems indicative of a compact soil/dust external layer. Both the apparently clean areas (similar to that shown in Figure 2a) and area covered by the compact layer (similar to that shown in Figure 2b) were analyzed by EDX and results are reported in Table 1. As shown, the clean areas mainly evidenced the presence of carbon and oxygen, with significant amounts of silicon, calcium, sulphur, aluminum, magnesium, potassium, and iron (total amount about 5.3 wt %). Moreover, the presence of inorganic contaminants was found more significant in the compact layers, for which the same element accounted for about 22.5 wt %. The same analysis performed on the milled sample MBW, evidenced a significant reduction of the inorganic component, for which the total amount of the same elements was found lower than 0.8 wt %. This can be explained taking into account that contaminants, mainly located at the surface of wooden pieces, after milling becomes slightly relevant with respect to the total mass of the organic fraction.

**Figure 3:**
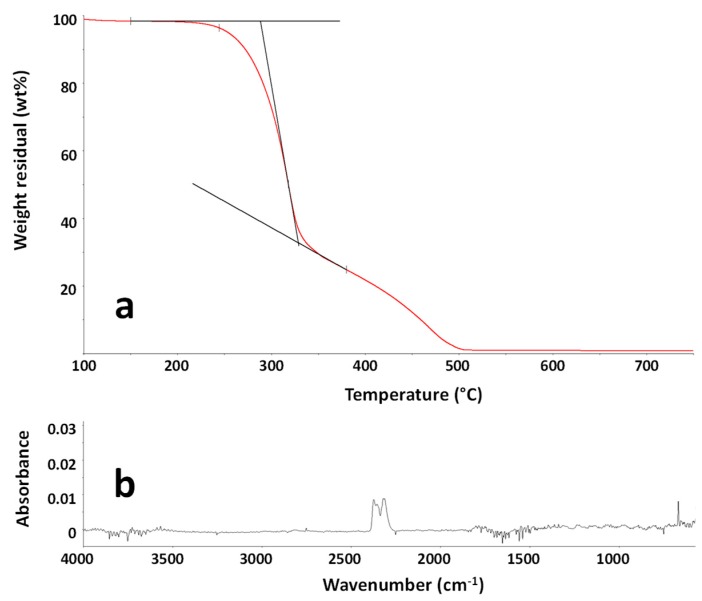
TG curve of the sample MBW in the temperature range 100–750 °C (**a**). FTIR spectrum of the evolved gases from the sample MBW at 260 °C during TG experiment (**b**).

To investigate the thermo-oxidative stability of the MBW sample and to determine the evolved gases during a thermal treatment in view of their recycling, EG analysis was performed through the above detailed TG-FTIR apparatus. The TG curve of the sample MBW is reported in Figure 3a. As shown, the degradation of the sample occurred in two steps, associated with the difference in degradation rate of the cellulose/hemicellulose components (less thermally stable) and the lignin, which in turn degrades slowly over a wide range of temperature [47,48]. To evaluate the processability of the sample as a filler in polymer composites, the gas evolution at the first stage of degradation, occurring close to the common processing temperatures of polymer composites, was considered relevant. In particular, the analysis of the TG curve showed that, after a weight loss due to water evaporation, occurring until about 120 °C, the MBW sample started to decompose at about 220 °C, with a 2 wt % weight loss occurring at about 240 °C. The FTIR spectrum of the evolved gases at 260 °C, typical carbon dioxide spectrum, is shown in Figure 3b. The analysis of the evolved gases demonstrated that until 320 °C, corresponding to the maximum degradation rate of the first degradation step, mainly carbon dioxide was emitted. This is consistent to what was reported in the literature for different wood samples [49], confirming that these MBW samples are suitable for the use as a filler in polymer composites due to the absence of harmful substances released up to about 220 °C.

#### 3.1.2. CDW Sample

**Figure 4:**
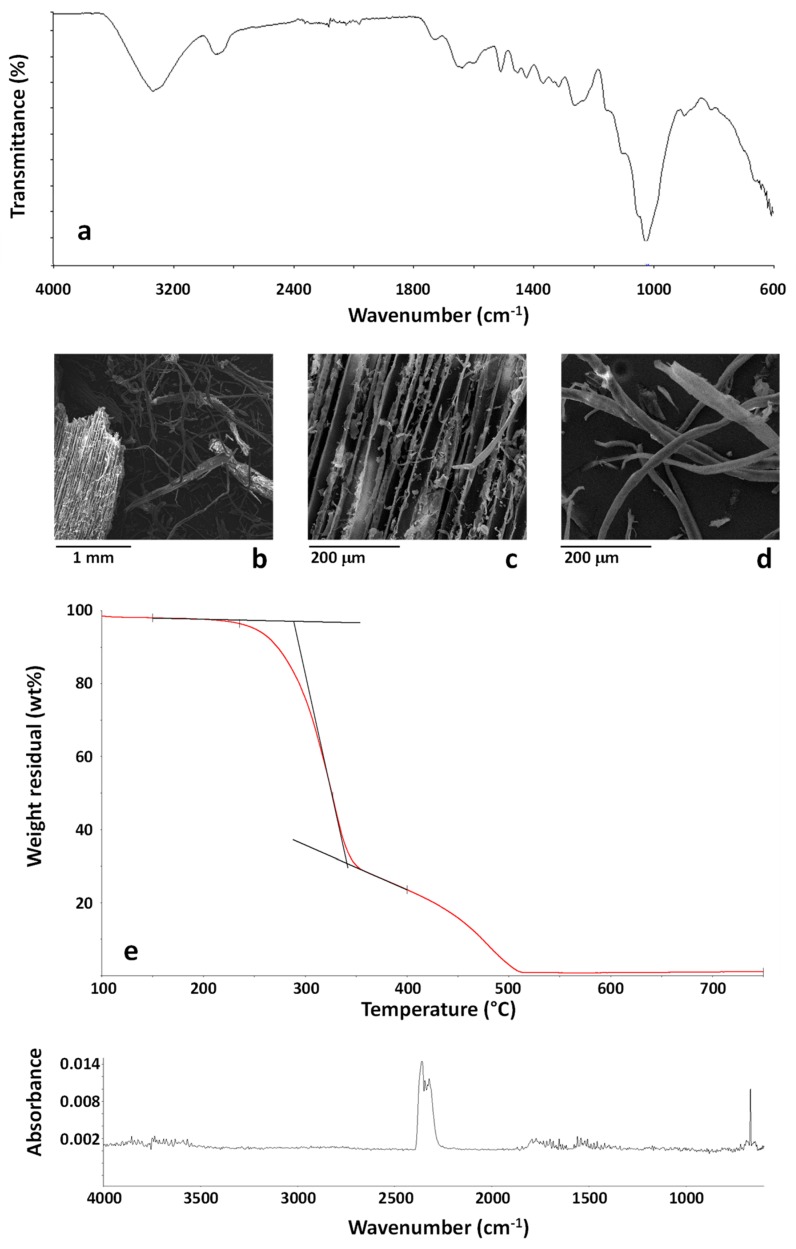
FTIR spectrum (**a**) and SEM images (**b**–**d**) of the CDW sample. TG curve of the sample CDW in the temperature range 100–750 °C (**e**). FTIR spectrum of the evolved gases from the sample CDW at 260 °C during TG experiment (**f**).

Using the same analytical protocol described for MBW, the sample CDW was first analyzed by ATR-FTIR. The spectrum of a CDW particle is reported in Figure 4a. The sample is almost homogeneous, with no significant differences amongst FTIR spectra collected on different CWD particles. FTIR analysis well demonstrates the lignocellulosic nature of all the samples, with the typical cellulose complex absorption band in the region 900–1200 cm^−1^ [17]. 

The wooden nature of the CWD samples was confirmed by SEM analysis, whose results are reported in Figure 4b–d. As shown CWD was constituted by wooden particles, such as that shown in Figure 4c, and relevant amounts of a partially defibrillated material, as shown in Figure 4d. Results of EDX analysis on this sample mainly indicated the presence of carbon (50.24 ± 2.69 wt %), and oxygen (49.19 ± 2.85 wt %) with silicon (0.10 ± 0.04 wt %), sulphur (0.10 ± 0.04 wt %), calcium (0.17 ± 0.04 wt %) and copper (0.18 ± 0.10 wt %) contaminants, with all these elements accounting for a total of 0.55 ± 0.26 wt %. Therefore, the amount of inorganic contaminants was not very relevant and the loose fibrils observed in Figure 4d can be identified as cellulosic material. The presence of copper should be taken into account in view of the recycling process as copper can catalyze the degradation of polyolefins [50].

TG-FTIR evolved gas analysis was carried out on the CDW sample using the same conditions detailed for the sample MBW. The TG curve of the CDW sample is reported in Figure 4e. As shown, the TG curve is very similar to that recorded for MBW, confirming the wooden nature of the CDW sample. A slightly higher residue at 750 °C is recorded for CDW (about 1.12 wt %). The analysis of first step of degradation shows that, after a weight loss due to water evaporation, occurring until about 120 °C, the CDW sample starts to decompose at about 220 °C, with a 2 wt % weight loss occurring at about 235 °C. Similarly to what was found for MBW, the analysis of the evolved gases from the CDW sample during combustion demonstrates that until 327 °C, corresponding to the maximum degradation rate of the first degradation step, mainly carbon dioxide is emitted. The FTIR spectrum of the evolved gases at 260 °C, typical carbon dioxide spectrum, is shown in Figure 4f. The obtained results confirmed that, similarly to what was evidenced for MBW, the analyzed CDW are suitable for the use as a filler in polymer composites due to the absence of harmful substances emitted up to about 220 °C.

#### 3.1.3. FW Sample

**Figure 5:**
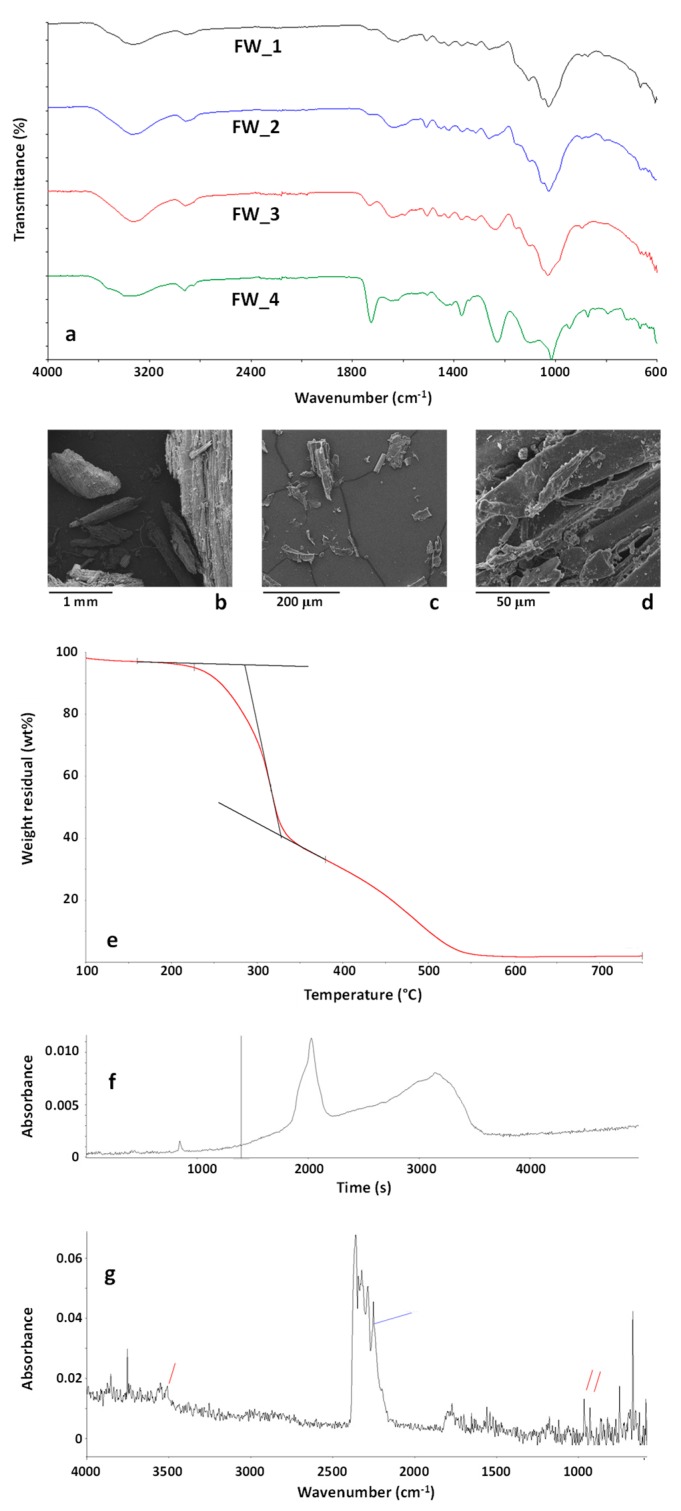
FTIR spectra of FW particles (**a**); SEM images (**b**–**d**) of the FW sample. TG curve of the sample FW in the temperature range 100–750 °C (**e**). Gram-Schmidt profile (**f**) and FTIR spectrum (**g**) of the evolved gases from the sample FW at 260 °C during TG experiment.

ATR-FTIR spectra of representative FW samples are reported in Figure 5a. FTIR analysis revealed that the sample was almost homogeneous, with small differences amongst FTIR spectra collected on different FW particles. Nevertheless, as shown in the FTIR spectrum FW_4 in Figure 5a, the presence of binders and/or faced layer typical of wooden panels was evidenced by the presence of a carbonyl absorption band.

The wooden nature of the FW samples was confirmed by SEM analysis, whose results are reported in Figure 5b–d. As shown, FW was mainly constituted by wooden particles of variable size (from a few mm to tens of µm) obtained from the grinding of different wood waste present in furniture, such as particleboards, plywood, fiberboards, and softwood. Results of EDX analysis on this sample mainly indicated the presence of carbon (48.13 ± 1.62 wt %), and oxygen (46.91 ± 0.92 wt %) with silicon (0.90 ± 0.16 wt %), sulphur (1.17 ± 0.44 wt %), chlorine (0.41 ± 0.08 wt %), potassium (0.48 ± 0.08 wt %), calcium (1.78 ± 0.30 wt %) and iron (0.21 ± 0.12 wt %) contaminants. As shown, the overall amount of inorganic contaminants was lower than 5 wt % and it would be compatible with the recyclability of the sample.

Results of TG-FTIR evolved gas analysis of the FW sample are reported in Figure 5e–g. Additionally for this sample (Figure 5e) a two-step degradation process was observed, with a higher residue at 750 °C (about 2 wt %) with respect to MBW and CDW. Despite to their similar wooden nature, the FW sample showed an anticipated degradation with respect to MBW and CDW, the sample starting to decompose at about 225 °C (about 2 wt % weight loss) and the maximum degradation rate of the first decomposition step occurring at 316 °C. 

The Gram-Schmidt profile of FW is reported in Figure 5f. The point corresponding to the temperature of 225 °C (about 2 wt % weight loss) is marked in the graph. The FTIR spectrum of the evolved gases corresponding to this point is reported in Figure 5g. As shown, apart CO_2_ absorption bands, new bands appeared, typical of isocyanic acid, HCNO, marked in blue, and NH_3_, marked in red. These emissions were already evidenced and analysed during pyrolysis and combustion of urea-formaldehyde resins [51]. The presence of these bands is well explained considering that furniture wastes are typically constituted by different types of wood boards containing urea-formaldehyde binders. To be also underlined is that these emissions were significant also at temperatures as low as 220 °C, very close to processing conditions of polymer composites. Therefore, if FW are considered for recycling as fillers in polymer matrices, processing conditions in terms of nominal processing temperature (depending on the type of polymer matrix) and possible friction-induced overheating phenomena should be carefully assessed and avoided by an appropriate design, to prevent harmful emissions that could affect the safety of the working environments and of the final products.

### 3.2. ELV Samples

**Figure 6:**
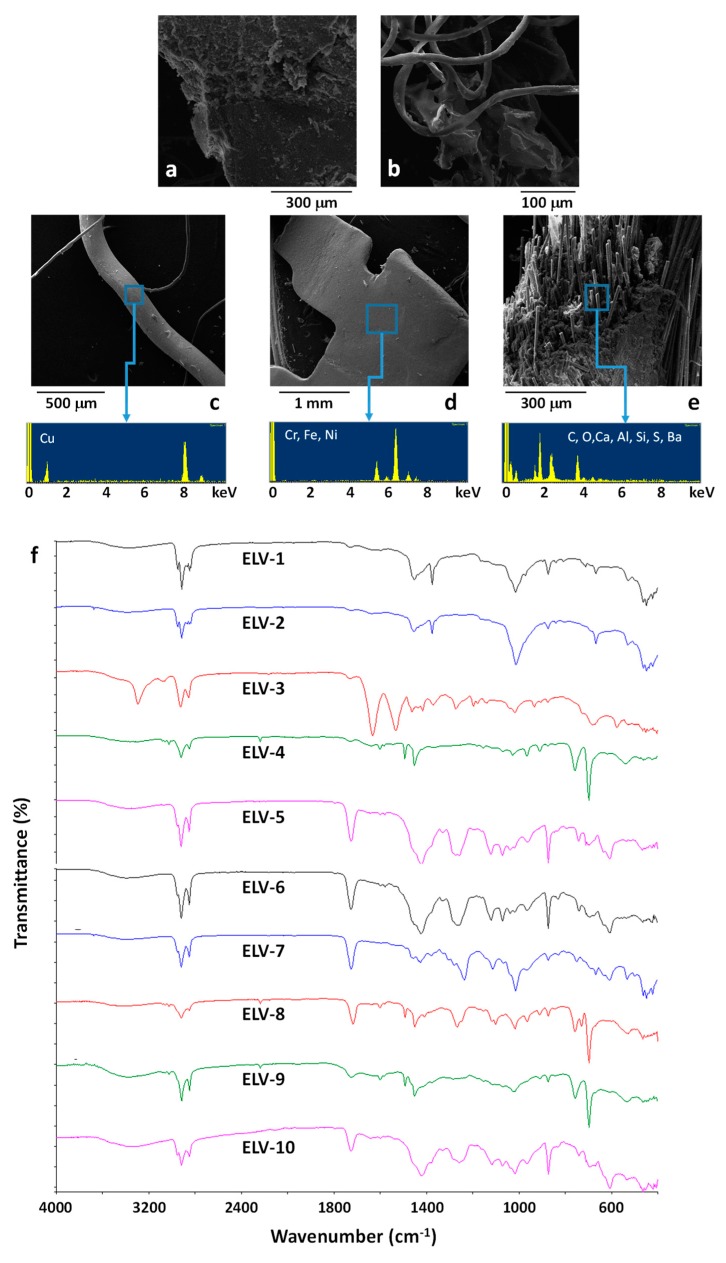
SEM images (**a,b**) and SEM/EDX results (**c,d,e**) of untreated ELV samples. ATR-FTIR of randomly selected ELV particles (**f**).

A preliminary, visual analysis of the untreated waste obtained by the shredding of the light fraction of EOL vehicles (ELV) indicated that the sample was mainly constituted by plastic particles with lateral dimension close to 3 mm containing a large number of impurities. SEM/EDX analysis (Figure 6a–e) confirmed the presence of polymer particles (Figure 6a) and polymer fibres (Figure 6b), mainly constituted by carbon and oxygen. Moreover, metal impurities were also evidenced, mainly constituted by copper wires and steel particles, as shown in the Figure 6c,d. Moreover, particles constituted by glass fiber reinforced composites were also found, containing typical elements constituting glass fibers, such as silicon aluminum and barium (Figure 6e).

To better investigate the contamination of ELV by inorganic or metal elements, a representative amount of ELV was thermally treated at 800 °C for 2 h, under air atmosphere, to remove most of the organic component. A residual weight of about 25 wt % was observed. EDX analysis performed on the powder obtained at the end of the thermal treatment, after mechanical homogenization, revealed the composition reported in Table 2. Results reported in Table 2 indicate that the main non-organic contaminants of the ELV sample were possibly glass fibers (as shown by the high presence of Si in the residual) and copper elements, while the presence of steel, randomly evidenced by SEM/EDX analysis on the ELV particles (Figure 6e), was almost negligible.

With the objective of identifying the nature of the polymer fraction constituting the ELV sample, several particles were randomly selected and characterized by ATR-FTIR analysis, see spectra reported in Figure 6f. FTIR spectra ELV-1 and ELV-2 demonstrate the presence of polypropylene (PP) [52]. Spectrum ELV-3 is typical of polyamide 6 [53]. Spectra ELV-4, ELV-8, and ELV-9 are typical of an acrylonitrile-butadiene-styrene resin [54]. Finally, spectra ELV-5, ELV-6, ELV-7, and ELV-10, with the typical bands at 1435 and 610 cm^−1^ (CH_2_ wagging and C-Cl stretching, respectively) and the ester C=O stretching bands at about 1730 cm^−1^, typical of common plasticizers, are indicative of plasticized polyvinylchloride (PVC) [31,55,56,57] that seems one of the main constituent of the polymer fraction of ELV.

A Soxhlet extraction was then performed on the ELV particles in chloroform [58] for 12 h to evaluate the amount of plasticizers and low molecular weight substances. The procedure gave a 21.0 wt % weight reduction after extraction, indicating the large presence of plasticizers (either from PVC either from other polymeric materials) and additives soluble in the selected solvent. 

**Figure 7:**
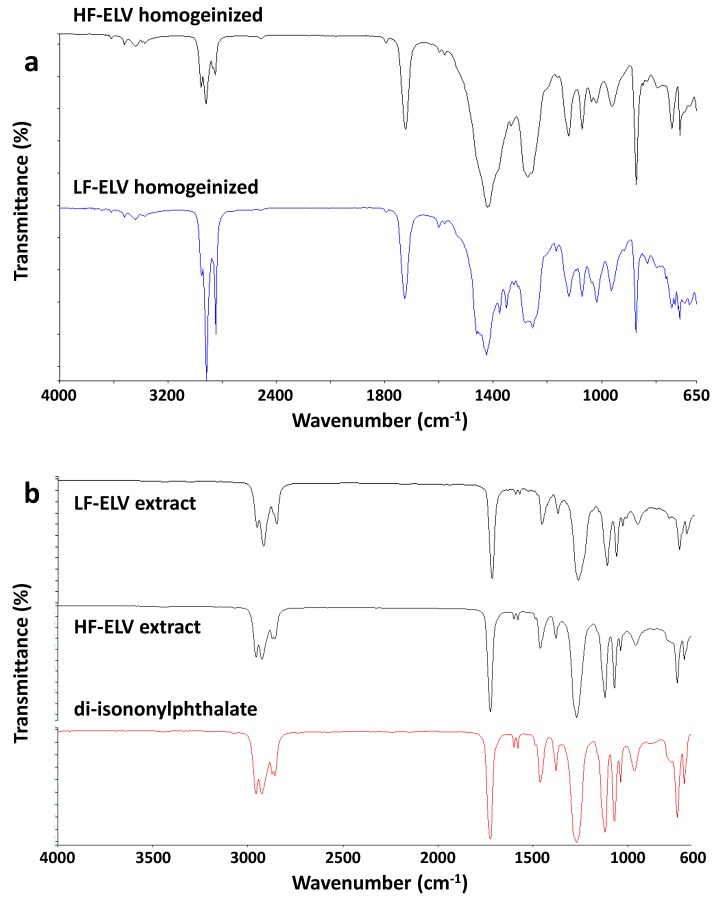
ATR-FTIR spectra of: (**a**) Homogeinized LF-ELV and HF-ELV samples; (**b**) extracts in chloroform from LF-ELV and HF-ELV samples and di-isononylphthalate reported for comparison.

As detailed in the experimental, an air separation procedure was applied to separate the ELV sample into two batches, denoted as LF-ELV (lighter fraction) and HF-ELV (heavier fraction). ATR-FTIR analysis was performed on the two batches LF-ELV and HF-ELV, after a pretreatment of homogenization performed in a melt mixing apparatus (Plastograph EC, Brabender GmbH & Co. (Duisburg, Germany), counter rotating blades, at 150 °C, 10 rpm, 10 min). ATR-FITR spectra of homogeneized LF-ELV and HF-ELV are reported in Figure 7a. Both samples showed the presence of the several absorption bands of plasticized PVC. Nevertheless, the high intensity of the bands at 2915 and 2848 cm^−1^ and the convoluted shoulder at 1471 cm^−1^ indicated the presence of polyethylene.

EDX analysis allowed to evaluate the Cl/C weight ratio in these samples. Cl/C weight ratio was found 0.25 for LF-ELV and 0.42 for HF-ELV, suggesting a lower amount of PVC in the LF-ELV sample. These results were confirmed by the application on these two batches of the same Soxhlet extraction procedure already applied for ELV. Soxhlet extraction gave 17.9 wt % weight reduction after extraction for LF-ELV and 25.1 wt % weight reduction after extraction for HF-ELV. The extracted phases were analyzed by ATR-FTIR, showing absorption bands typical of phthalate plasticizers. Indeed, in Figure 7b ATR-FTIR spectra of extracted from LF-ELV, HF-ELV are reported together with the ATR-FTIR spectrum of an alkyl phthalate, a common PVC plasticizer. Therefore, the evidence that LF-ELV showed the lower Cl/C ratio and the lower amount of extracted phase, indicated that the batch LF-ELV is less rich in PVC and possibly more contaminated by other polymeric materials, demonstrating the partial efficiency of the adopted separation procedure to separate the polymer fractions on the base of their density.

**Figure 8:**
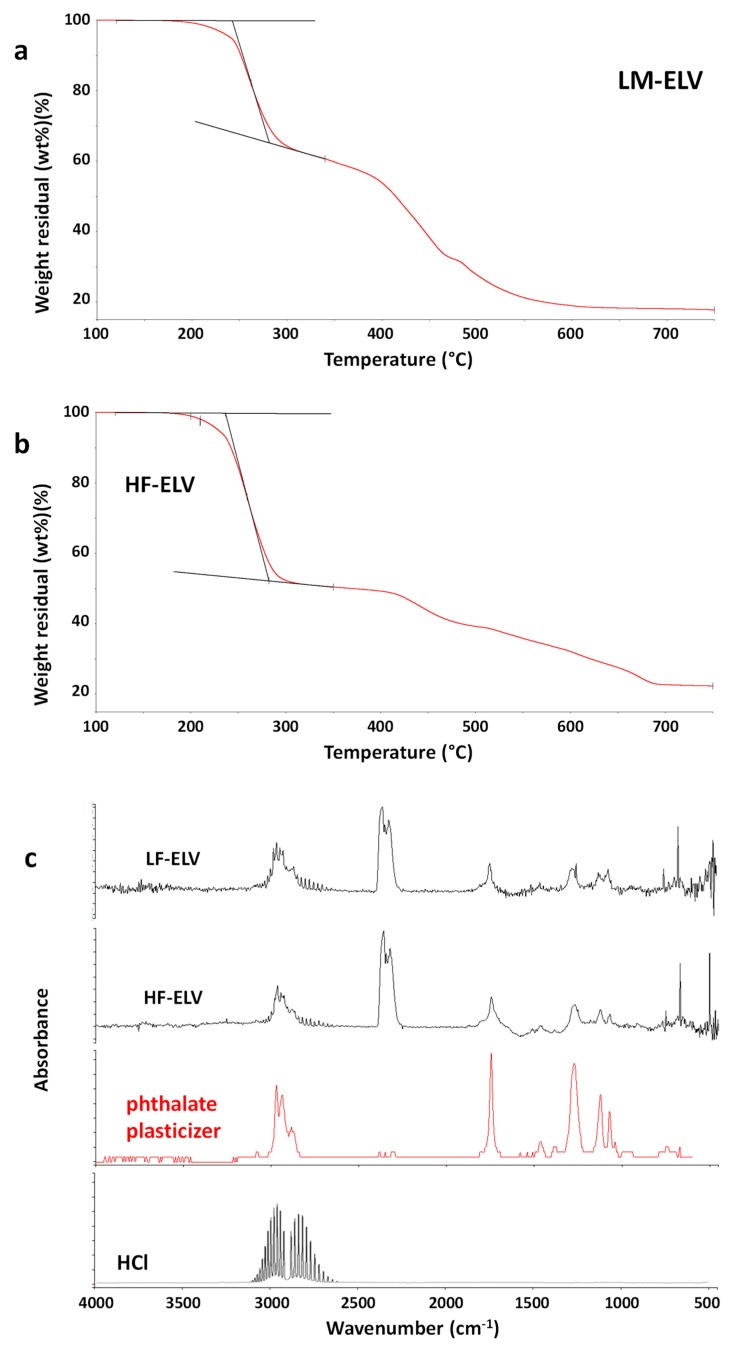
TG curves of the samples LF-ELV (**a**) and HF-ELV (**b**) in the temperature range 100–750 °C. FTIR spectra of evolved gases from LF-ELV and HF-ELV samples at 200 °C under air flow. The gas spectra of an alkylphthalate plasticizer and hydrochloric acid are reported for comparison (**c**).

TG-FTIR evolved gas analysis of the HF-ELV and LF-ELV samples was performed. TG curves are reported in Figure 8a,b. As shown, the TG profiles for the samples were found very similar. A weight loss of about 2 wt % was recorded at 200 °C either for LF-ELV either for HF-ELV. The first degradation step was more relevant for the sample HF-ELV, containing a higher amount of PVC fraction with respect to LF-ELV, thus suggesting that this degradation step is attributable to one of the components of plasticized PVC. FTIR spectra of evolved gases from both samples at 200 °C are reported in Figure 8c. As shown, CO_2_ was emitted from both samples, together with a component whose FTIR spectrum is very similar to a phthalate plasticizer, whose gas phase spectrum is reported by comparison. Moreover, the presence of hydrochloric acid is also evidenced by the presence of the absorption bands in the 2600–3200 cm^−1^ region. Therefore, for both LF-ELV and HF-ELV emissions were significant also at relatively low temperatures, starting to be detectable at about 190 °C. These results clearly indicate that the recycling of plastic fractions recovered from ELV should take into account the large presence of PVC and thus it should be carefully designed and investigated to avoid harmful emissions during processing. Indeed, the wrong selection of the processing conditions could lead to the emission of harmful gases, mainly hydrochloric acid and phthalates, both very dangerous by inhalation [59,60,61].

### 3.3. WTB Sample

**Figure 9:**
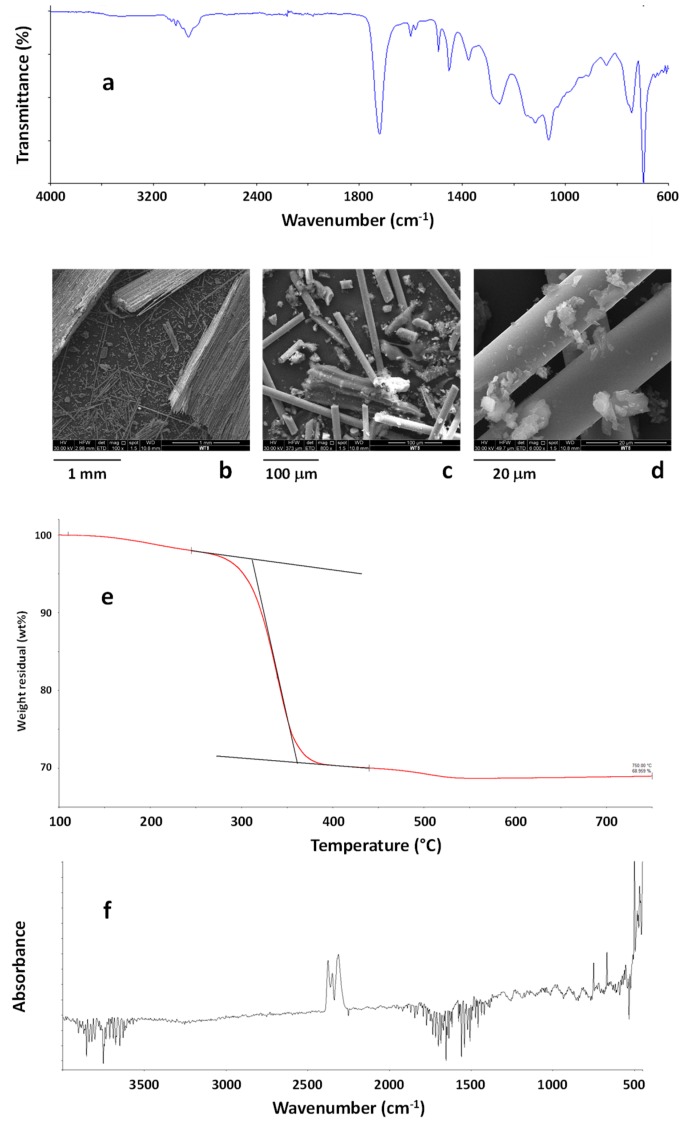
ATR-FTIR spectrum of wind turbine blade (WTB) waste (**a**). SEM images of WTB waste (**b, c, d**). TG curves of the WTB sample in the temperature range 100–750 °C (**e**). FTIR spectra of evolved gases from WTB waste at 280 °C under air flow (**f**).

WTB wastes were the third class of EOL materials analyzed. ATR-FTIR spectroscopy analysis was carried out on WTB powders and results are reported in Figure 9a. ATR-FTIR spectra revealed that the sample was quite homogeneous, and mainly constituted by a vinylester resin [62] reinforced with glass fibers. Representative SEM images of the WTB sample are reported in Figure 9b–d. SEM micrographs clearly showed the presence of isolated glass fibers as well as fiber bundles still embedded in the polymer matrix. EDX analysis was also performed on the sample and results are reported in Table 3. In this table, the quantification of carbon has been omitted to better identify the presence of other elements. As shown, several elements constitute the sample typical of glass fibers. Comparing the results of EDX with data reported in literature [63], glass fibers have been identified as E-glass.

TG profiles (Figure 9e) indicated a very high residual weight at 750 °C (69 wt %), mainly due to the presence of glass fibers, although the recorded residual weight is not able to indicate the exact amount of the inorganic component in the composite due to the uneven distribution of the polymer phase and the low amount of material that can be investigated by TG analysis. Moreover, a weight loss of about 2 wt % was recorded at 245 °C whereas the maximum degradation rate occurred at 339 °C. TG-FTIR also revealed that mainly water and carbon dioxide were evolved during the first degradation stages, up to about 270 °C, whereas other organic components started to be evolved during the main degradation step, starting at about 280–300 °C. This result indicated that the analysed grinded WTB wastes can be safely used as rigid fillers by mixing them in thermoplastic matrices, for instance polyolefins, at the typical temperatures used for their compounding. Nevertheless, as shown by morphological analysis, effective precautions would be required in the working environments because of the presence of free fiberglass in the WTB sample, and a careful analysis of the new materials produced using recycled WTB wastes should be performed to exclude the release of fiberglass during the use of the realized products.

## 4. Conclusions

Wooden wastes, plastic fractions from automotive shredded residues, and glass fiber reinforced composites obtained from dismantled wind turbines blades are relevant classes of wastes currently under investigation for their possible recycling in polymer composites realized for different applications, including automotive components, furniture, and construction elements. 

In this work, the above identified classes of wastes were analyzed in view of their possible recycling. In particular, an analytical protocol was applied, based on FTIR analysis, SEM/EDX analysis, and TG analysis in air flow, for the identification of the main constituents and contaminants of the waste samples. TG analysis combined to FTIR of the evolved gases was very useful to evaluate the possible evolution of harmful substances during high temperature treatments typical of the processing conditions of thermoplastic polymers and composites. 

Results showed that all the investigated classes of wastes are very complex and heterogeneous. The main components and contaminants identified in wooden wastes, plastic fractions from automotive shredded residues, and glass fiber reinforced composite are resumed as follows. 

Wood wastes. Main constituent: Mixed wood species; contaminants: Plastics, metals, paper, inorganic contaminants (soil, dust), binders (UF resins, MF resins).

ELV. Main constituent: PVC; contaminants: Non-PVC plastics, plasticizers (alkyl phthalates), metals, inorganic contaminants, GFRP.

WTB. Main constituent: GFRP; contaminants: No extraneous substances were detected.

The revealed contaminants represent possible serious obstacles toward a safe and cost-effective recycling of the selected end-of-life materials.

In particular, depending on their origin, wood wastes showed different levels of contamination. The heavy fraction of wood from municipal bulky waste (MBW) mainly showed the presence of soil and dust contamination, whose relative amount was very low and did not preclude the direct recycling of the wood particles as fillers in polymer composites after appropriate grinding.

Construction and demolition wastes (CDW) showed similar level of contamination, although in this case some metal contaminants (such as copper) would be taken into account in view of the recycling of CDW wooden particles as fillers in polymer composites after appropriate grinding.

A different situation must be underlined for furniture wastes (FW), constituted by wooden particles obtained by grinding of different products typically used in the furniture sector, such as particleboards, plywood, fiberboards, and solid/soft wood. Most of these products contain binders whose thermal stability is only partially compatible with their recycling as fillers in polymer composites. For these materials, the evolved gas analysis showed the emission, during thermal treatments, of different substances, such as isocyanic acid and ammonia. These emissions were significant also as temperatures close to processing conditions of polymer composites, indicating that the reprocessing of FW must be carefully designed to prevent harmful emissions that could affect the safety of the working environments and of the final products.

For what concerns the analyzed ELV samples, they are constituted by shredded plastic particles containing different amounts of metal impurities, depending on the separation technology adopted. The plastic fraction of these samples is mainly constituted by plasticized PVC, mixed to other polymers, such as polyolefins and ABS resins. Thus, the direct reprocessing of the ELV samples is not able to produce a recycled material with satisfactory properties, mainly because of the presence of several immiscible polymer fractions. Moreover, the eventual processing of ELV wastes must be carried out at relatively low temperatures to prevent emission of harmful substances such as phthalates and hydrochloric acid. For this class of wastes, more effective sorting technologies should be developed to separate and thus reprocess the PVC fraction.

Finally, the analysis of a particular class of wastes, namely shredded wind turbine blade (WTB) samples, revealed that they are mainly constituted by fiberglass reinforced thermosetting polymer particles, containing large amounts of free fiberglass fragments produced during grinding. WTB wastes can be effectively recycled through appropriate technologies, such as their embedding as rigid fillers in thermoplastic matrices for the realization of high performance composites. In this case, effective precautions must be planned in the working environments because of the presence of free fiberglass in the WTB sample, and a careful analysis of the final materials realized would be necessary to exclude the occurring of releasing phenomena of fiberglass during the life of the realized products.

## Figures and Tables

**Table 1:** Results of energy dispersive X-ray (EDX) analysis (wt %) on the samples MBW-Px and MBW.

Element	MBW-Px Clean Surfaces ^1^	MBW-Px Compact Layers ^2^	MBW
C	48.04 ± 2.34	27.7 ± 1.41	50.58 ± 0.92
O	46.67 ± 1.04	49.7 ± 2.08	48.67 ± 0.96
Mg	0.17 ± 0.10	0.52 ± 0.16	0.04 ± 0.01
Al	0.73 ± 0.35	2.55 ± 0.63	0.10 ± 0.06
Si	1.92 ± 0.89	7.02 ± 1.73	0.14 ± 0.08
S	0.81 ± 0.05	3.39 ± 0.10	0.08 ± 0.05
K	0.17 ± 0.03	0.84 ± 0.09	0.07 ± 0.05
Ca	1.27 ± 0.07	6.65 ± 0.09	0.29 ± 0.12
Fe	0.22 ± 0.05	1.58 ± 0.18	0.03 ± 0.02

^1^ Morphology similar to those shown in Figure 2a. ^2^ Morphology similar to those shown in Figure 2b.

**Table 2:** Results of EDX analysis on the ELV residual after thermal treatment at 800 °C in air and homogenization of the recovered fraction.

Element	Amount (wt %)
C	26.98 ± 4.36
O	50.70 ± 2.61
Na	0.59 ± 0.02
Mg	4.20 ± 0.59
Al	2.00 ± 0.01
Si	7.80 ± 1.21
S	0.72 ± 0.01
Cl	1.10 ± 0.34
K	0.12 ± 0.01
Ca	3.27 ± 0.62
Ti	0.33 ± 0.05
Cr	0.02 ± 0.01
Fe	0.27 ± 0.04
Cu	1.03 ± 0.05
Zn	0.62 ± 0.02
Ba	0.26 ± 0.01

**Table 3:** Results of EDX analysis on WTB sample (carbon omitted).

Element	Amount (wt %)	Element	Amount (wt %)
C	omitted	Cl	0.597 ± 0.219
O	70.857 ± 0.383	K	0.207 ± 0.015
Na	0.457 ± 0.077	Ca	6.806 ± 0.247
Mg	0.394 ± 0.011	Ti	0.180 ± 0.089
Al	4.469 ± 0.039	Fe	0.698 ± 0.056
Si	14.954 ± 0.238	Ni	0.007 ± 0.004
S	0.307 ± 0.199	Cu	0.067 ± 0.021
